# Hepatic hydatid disease: four case reports

**DOI:** 10.1186/1757-1626-2-58

**Published:** 2009-01-15

**Authors:** Attef M Elshazly, Manar S Azab, Samar N ElBeshbishi, Hany M Elsheikha

**Affiliations:** 1 Department of Parasitology, Faculty of Medicine, Mansoura University, Mansoura 35516, Egypt; 2 The School of Veterinary Medicine and Science, The University of Nottingham, Sutton Bonington Campus, Loughborough, Leicestershire, LE12 5RD, UK

## Abstract

We report four cases who were referred to Mansoura University Teaching Hospital, Egypt suffering from abdominal pain and gastrointestinal manifestations. The patients' history was unremarkable, except that they had contact with dogs and live in rural communities. Laboratory findings showed peripheral blood eosinophilia, leucocytosis, and elevated liver enzymes. Serological tests were positive in three cases. Ultrasonography showed well-circumscribed cystic masses in the liver. Diagnosis of hydatid cysts was confirmed by computed tomography (CT). Surgical treatment along with chemotherapy was performed and all patients recovered well. The results of these cases support the notion that CT scan can led to increased clarity, regarding surgical management, because of discordance between radiographic and laboratory findings.

## Background

Human cystic echinococcosis (CE)/hydatidosis is a dog-borne zoonoses caused by infection with the larval stage of the dwarf tapeworm of the genus *Echinococcus*. *E. granulosus *and *E. multilocularis *are the most important members of the genus in respect of their public health importance and their geographical distribution [1]. The life cycle of *Echinococcus *is indirect and involves two hosts, one definitive carnivore host and the other intermediate herbivore host [2]. The problem arises when humans act as an accidental intermediate host and ingest viable oncosphere-containing eggs, which have been shed in the faeces of the definitive host. The oncospheres invade the intestines, enter the vasculature and develop into hydatid cysts in any organ or tissue, where a variety of symptoms can be produced. However, the liver acts as the first filter for hydatid larvae, making it the most commonly affected organ followed by lung [1].

The diagnosis of CE is based on the patient's history, clinical findings, haematological and serum biochemical profiles, and serological testing, which may be negative in 10% to 20% of cases [3]. Efforts to improve diagnostic accuracy have led to integration of a range of imaging techniques into the diagnostic armamentarium [1,4]. The radical surgical removal of the cystic lesion remains the mainstay of treatment with a high success rate [1,4]. Chemotherapy, with benzimidazole compounds has also been used with some success to sterilize the cyst, decrease the chance of anaphylaxis, and reduce the complications and recurrence rate post-operatively. In recent years, a third treatment option was introduced (PAIR, puncture, aspiration, injection, and re-aspiration) and is indicated for patients who cannot undergo surgery [5,6].

Despite efforts to control CE in many parts of the world the disease continues to exert a heavy burden on human health. And, in a number of countries, including Egypt, CE is re-emerging as a major public health issue, with potentially life-threatening complications [7]. Herein, we report four cases of CE from Egypt.

## Case presentation

The four patients described in this report were seen and evaluated in the Department of Internal Tropical Medicine, Mansoura University Teaching Hospital, Egypt between January 1, 2007 and September 30, 2007 for complaints of abdominal pain and gastrointestinal (GI) manifestations. Patients were interviewed and examined by a physician, after which venous blood samples were drawn from an antecubital vein and were processed immediately. Blood samples were assessed for some haematological parameters, including red blood cell count (RBC), white blood cell count (WBC), eosinophil (%) and hemoglobin (Hb) content. Analysis was done using an Automated Hematological Analyzer, Sysmex TM-K-1000 (TOA Medical Electronics Company Ltd., Kobe, Japan). Sera were used to determine some key biochemical parameters. These included: (1) total serum bilirubin using commercial kit (Quimica Clinica Aplicada, Spain), and (2) activity levels of alanine aminotransferase/aspartate aminotransferase (ALT/AST) and alkaline phosphatase (ALKP), markers of hepatocellular damage according to Reitman-Frankel colorimetric transaminase procedure.

The serum samples were tested by the echinococcosis haemagglutination test (IHA) Fumouze^® ^(Laboratoires Fumouze, Levallois-Perret, France) and ELISA (F-Form, Maxisorp, Nunc, Roskilde, Denemark). Pre-operative diagnosis of hepatic cystic lesions was made ultrasonically and confirmed by a computer tomography (CT) scan. After completion of this evaluation process surgical consultation was sought. All four patients were successfully managed surgically and all were asymptomatic at the two years follow-up (Table 1). This research was approved by the Mansoura University Faculty of Medicine Ethics Board.

**Table 1:** Pertinent details of hepatic hydatid cases.

	Case 1	Case 2	Case 3	Case 4
Gender	Female	Male	Male	Male
Age (Years)	22	11	37	43
Occupation	Student	Farmer	Farmer	Shepherd
Residence	Suburban	Rural	Rural	Rural
Dog contact	No	Yes	Yes	Yes
Symptoms	Abdominal pain, nausea	Indigestion, easy fatigability	Generalized abdominal pain, nausea, vomiting, diarrhoea	Pain in right hypochondrium, nausea, vomiting, weight loss
Physical examination	Hepatomegaly	Normal	Normal	Hepatomegaly
Hemoglobin (g/dl)	12	13.2	14	14.1
RBCs (106/ml)	3.9	4.4	4.8	4.5
WBCs (103/ml)	8.4	12.7	6.6	19.2
Eosinophils (%)	8	18	4	17
Bilirubin (mg/dl)	1.7	2.2	0.9	1.2
ALT (U/ml)	58	34	22	41
AST (U/ml)	74	61	34	36
ALKP (U/L)	18	22	16	11
Serology (test)				
IHA	Positive	Negative	Positive	Positive
ELISA	Positive	Negative	Positive	Positive
Maximal diameter (cm)	6	14	5	6
Cyst type	III	I	I	I
Concurrent locations	Spleen	--	--	--
Surgical Treatment	Complete pericystectomy	Complete pericystectomy	Complete pericystectomy	Complete pericystectomy
Postoperative complications	--	--	--	--

A provisional diagnosis of hydatid disease was made based on the clinical manifestations, haematological and biochemical parameters, and serological tests. The diagnosis was confirmed by radiological imaging. The important demographics and clinical features, laboratory findings and therapeutic intervention in these patients are summarised in table 1. The mean age of the patients was 28.3 (range 11–43) years. Of the four patients, three were male and one was female. Three of the four cases had frequent contact with dogs and farm animals and live in agricultural sheep-grazing area which is in line with the general epidemiology of hydatidosis. All patients were non-alcoholic and non-smoker. Presenting signs and symptoms for the 4 patients are listed in Table 1. All of them had intermittent colicky pain associated with nausea and vomiting. Except one patient (case one) who has a history of acute symptoms lasting 1–2 months, their mild symptoms began more than one year ago.

Haematological studies revealed leucocytosis in all cases and eosinophilia in three cases. RBC count and Hb content were within normal range. Total serum bilirubin was elevated in three cases and was within normal range in one case (case three). The biochemical serum level of ALT was significantly raised in one patient (case one) and the AST level was increased in cases one and two. Analysis of the liver enzyme ALKP revealed abnormal increase in three cases and normal level in case four. Serologic echinococcal tests are valuable when they are positive, but one of the four hepatic hydatidosis cases gives a false negative. Although serology tests like ELISA and IHA can help make the diagnosis, complete reliance on them is not recommended.

**Figure 1:**
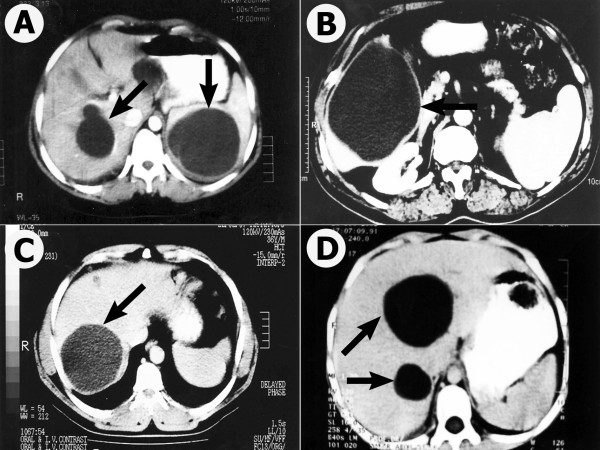
**Photographs of transverse computed tomography scan through the liver showing cystic hydatid lesions**. Figs. A, B, C, and D refer to case one to four, respectively. Arrows indicate the hydatid cyst.

Radiographic evaluation confirmed the variable nature of the clinical hydatid disease that has been previously reported [8]. Hepatic radiograph appearance was variable and revealed abnormal findings (Fig. 1). In case one (Fig. 1A) liver had a relatively large size, normal echopattern, but, with two well defined sonolucent cysts with thin internal septae. Liver in case two (Fig. 1B) showed average size with a well defined hypodense non-enhancing hydatid cystic lesion and is seen affecting segment V and VI. It also compressed the gall bladder, displaced the patent non-dilated portal vein (PV). No other focal lesions and no dilatation of the intra or extra-hepatic bile ducts were noticed. Liver in case three (Fig. 1C) had a large well-defined hypodense hydatid cyst involving the postro-superior segment of the right lobe (segment VII). The liver of case four (Fig. 1D) was enlarged with smooth contour and uniform enhancing parenchyma with normal patent PV. Multiple variable-sized cystic lesions are seen scattered in both hepatic lobes. Further investigations ruled out coexistent hydatid disease in other organs except spleen in case one.

## Discussion

From a clinical point of view, clinical manifestations of hydatidosis in humans are variable, most patients seem to tolerate the infection for extended periods without any symptomatology, or they may suddenly show dramatic and acute symptoms [8]. All four patients made an excellent recovery following surgical excision of the entire cystic masses. Adjunctive Albendazole chemotherapy 10 mg/kg/day was prescribed for 4–6 weeks before surgery to sterilise the cyst and for 2 months post-operatively to reduce the recurrence rate. At the 24-month follow-up after surgery, the patient remained free of symptoms and continued to do well. This result is consistent with other studies that found that CT scan has become an extremely useful and valuable diagnostic tool in the management of patients with hydatidosis [1,9]. Accuracy would have been enhanced by concomitant magnetic resonance imaging (MRI). But, MRI was not performed in this study, though we recognise that these might have been helpful [10].

As frequently as it occurs, hydatidosis can sometimes be a diagnostic quandary. With the concern that, left untreated, even solitary hydatid cyst in critical organ(s) will likely progress to grave and life-threatening illness, surgeons have historically coped with diagnostic uncertainty by accepting high negative surgical intervention rates as an alternative to risking disease progression. Taken haematological, biochemical, serological and imaging findings into account, some recommendations for more accurate and sensitive detection of clinical cases, regarding surgical management, can be made: (1) diagnosis is best accomplished by a combination of imaging, serologic, and laboratory investigations; (2) radiographic examination must be considered for patients in whom hydatidosis is a potential diagnosis; and (3) patients with equivocal history, physical findings, or laboratory results should not be considered negative unless there are no obvious positive radiographic findings.

Despite major advances in our understanding and treatment of hydatidosis over the past two decades, control of this zoonotic disease remains a challenging endeavour. Fortunately, because of the high risk of infection, the high morbidity rate and the unpredictable outcome of hydatid infection, there is an increasing realization in international health agencies that hydatidosis is an important disease that causes life-threatening morbidity. Like many diseases, prevention is the key to control, and we know that simple changes in social habits and hygiene can prevent infection and disease in humans. Also, chemoprophylaxis is an effective measure to protect humans living in endemic or hyperendemic areas of hydatid infection.

In conclusion, we stress the importance of maintaining a high level of clinical awareness that hepatic hydatidosis continues to be a clinical problem especially in endemic regions of the world and travellers to these areas should be made aware of the need to take on extra-measures to avoid hydatid disease. In endemic areas like Egypt any cystic enlargement of soft tissue should raise the suspicion of clinicians of hydatid disease. Serologic tests, ultrasonography, and CT should be performed before any invasive procedure.

## Competing interests

The authors declare that they have no competing interests.

## Authors' contributions

AME diagnosed and managed the cases with full responsibility. MSA provided data and accompanying images. SNE analyzed and interpreted the patients' data. HME was the general coordinator of the cases and was the major contributor in writing the manuscript. All the authors read and approved the final manuscript.

## Consent

Written informed consent was obtained from the patients for publication of this report and accompanying images. Copies of the written consent are available for review by the Editor-in-Chief of this journal.
